# More than a loading control: actin regulation in aging

**DOI:** 10.18632/aging.204746

**Published:** 2023-05-18

**Authors:** Maxim Averbukh, Gilberto Garcia, Ryo Higuchi-Sanabria

**Affiliations:** 1 Leonard Davis School of Gerontology, University of Southern California, Los Angeles, CA 90089, USA

**Keywords:** actin, aging, cancer, senescence

Aging is a complex phenomenon, typically characterized by a chronological, progressive loss of cellular and physical function. This general deterioration of cellular processes increases the risk for a variety of chronic diseases including cancer, heart disease, and Alzheimer’s disease (AD), which together increases the vulnerability to death. Among the cellular processes that decline in function during aging, stress response pathways have been shown to be integral for healthy aging. Regulatory stress responses function to protect organisms from damage associated with stress exposure and their breakdown can contribute to several aging phenotypes [1].

Organelle-specific stress responses have evolved to preserve homeostatic function of each specialized organelle compartment within eukaryotic cells. These include the cytosolic heat shock response (HSR) and the unfolded protein responses of the mitochondria (UPR^mt^) and endoplasmic reticulum (UPR^ER^), all of which contribute to homeostatic function of their designated organelle and have implications in longevity [1]. Despite major efforts in this field, the machineries dictating homeostatic function of the actin cytoskeleton during stress and aging are poorly understood. The actin cytoskeleton is a complex, dynamic network of protein filaments that provide structural support and shape to cells and has been implicated in many physiological age-related changes. For example, in multiple model systems, cytoskeletal form and function has been shown to decline with age, which can directly impact nutrient sensing and aging in *S. cerevisiae* [2] and thermotolerance and longevity in *C. elegans* [3]. Mechanistic function of the cytoskeleton is also important in mammalian systems, as dysfunctions in actin are implicated in age-associated diseases, such as Alzheimer’s Disease (AD): Cofilin, a major actin-binding protein, has been shown to have an impact on several major signature of AD including synaptic dysfunction, β-amyloid plaques, hyperphosphorylated tau, cofilin-actin rods, and Hirano bodies [4].

Despite the implications of the actin cytoskeleton contributing to aging physiology and disease, little is known about actin regulation throughout an organism’s lifespan. To date, there are two known “master” regulators of actin function: heat shock transcription factor-1 (HSF-1), and serum response factor 1 (SRF1). HSF-1 is an evolutionary conserved transcription factor vital for stress resistance and lifespan regulation. HSF-1 has been shown to regulate cytoskeletal function during aging and heat stress in *C. elegans*, however, investigation of HSF-1 transcriptional targets revealed only a small number of actin-regulatory genes [3]. SRF1 is also a highly conserved transcription factor implicated in cell migration, growth, and differentiation and vital for survival, with SRF’s primary function in cytoskeletal dynamics being linked to cell migration [5]. There are a few studies that correlated SRF1 function with aging in skeletal muscle cells, but a direct link to SRF and longevity is lacking.

Therefore, to identify additional conserved regulators of the actin cytoskeleton, we performed an unbiased, cross-species screens [6]. Specifically, we performed a whole-genome CRISPR-Cas9-mediated knockout screen in human fibroblasts, followed by an *in vivo C. elegans* RNAi screening to identify genes required for survival to actin stress. A number of targets were identified from the consecutive screens; however, *bet-1* was the only gene that showed correlations with lifespan in *C. elegans.* Specifically, *bet-1* knockdown resulted in shortened lifespan, while overexpression was sufficient to drive longevity. *bet-1* is a conserved (BRD4 in mammals) double bromodomain protein recognized for its role in cell fate determination with some links to actin function. Perhaps most importantly, BRD4 has been implicated in cancer aggression through promoting angiogenesis, a process highly dependent on actin [7], making *bet-1*/BRD4 a highly promising candidate for understanding the mechanistic role of actin during aging.

On a physiological level, our study found that BET-1 drives organismal health and longevity by promoting stability of muscle and intestinal actin, which maintained muscle motility and gut barrier function at advanced age. On a molecular level, this study placed BET-1 downstream of MYST histone acetyltransferases, which acetylate specific lysine residues on histones to allow for recruitment of BET-1 to chromatin. While our study did not identify mechanistically how BET-1 promotes actin health, transcriptome analysis revealed that overexpression of *bet-1* induces expression of actin regulatory genes. Moreover, the increased stability of actin is required for the beneficial effects of *bet-1* overexpression on organismal health and longevity.

**Figure 1:**
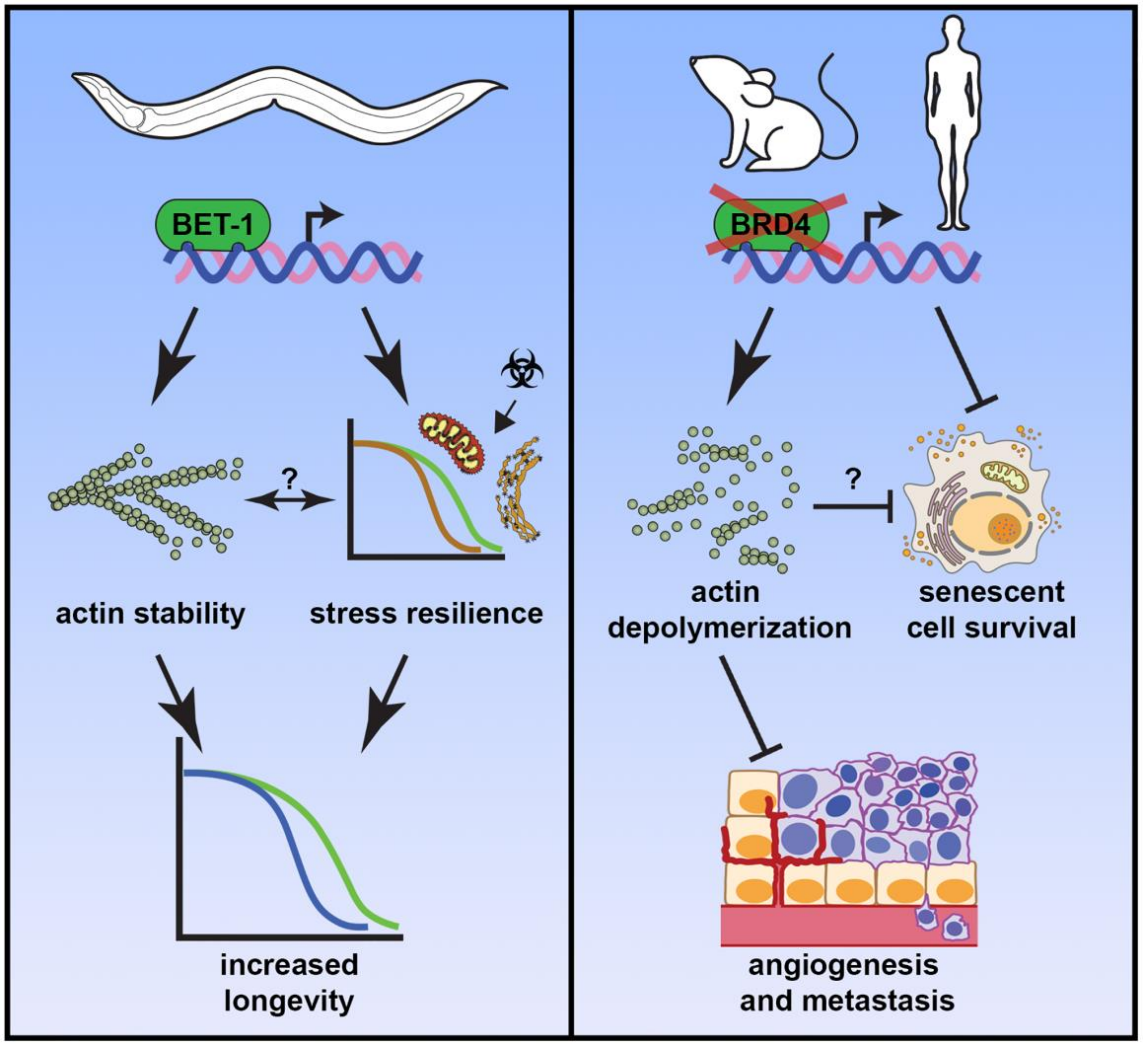
**BET-1/BRD4’s role in regulation of actin stability impacts aging and age-related diseases.** Left: In *C. elegans*, BET-1 can induce transcription of actin regulatory genes and promote stress resilience to promote longevity. While increased actin stability and stress resilience are both correlated with increased *bet-1* expression, whether increased actin stability directly impacts stress resilience or vice versa are still unknown. Right: In mammalian cells increased BRD4 activity can be detrimental by promoting senescent cell survival and increased cancer aggression. Inhibition of BRD4 activity results in destabilization of F-actin filaments, which can prevent angiogenesis and metastasis of cancer cells and is correlated with decreased senescent cell survival, although whether the decreased stability of actin is the direct cause of senescent cell death is still unknown. These data suggest that BRD4 can be a viable therapeutic target both for its anti-cancer and senolytic properties.

In addition to impacting longevity, *bet-1* overexpression increased stress resilience. Specifically, *bet-1* overexpressing animals exhibit increased resilience to mitochondrial, oxidative, and endoplasmic reticulum stress, with part of this resilience likely being driven by transcriptional changes that increase canonical stress responses like the UPR^ER^ [6]. This study primes BET-1 as a potential regulator of stress resilience and an important future study would be to determine whether the increase in stress resilience is due to an increase in actin stability or an unrelated mechanism. Indeed, previous studies have shown that increased actin stability is associated with an increased tolerance to thermal stress [3]. Finally, it is worth investigating whether a true “actin cytoskeletal stress response” (ACSR) exists whereby in response to stress, actin integrity can be maintained as a mechanism to drive resilience and organismal health. An exciting area of research can be to investigate whether a BET-1/BRD4 driven ACSR – possibly in coordination with other stress regulators – can drive overall stress resilience and longevity (Figure 1, left).

Although *bet-1* overexpression has a significant effect on lifespan, it has potential limitations when it comes to human health. The human ortholog BRD4 has pro-cancer properties, and it is commonly used as a target for anti-cancer therapy [7]. This is not a rare phenomenon in biology: pro-longevity pathways are often overlapping with pro-survival pathways, which can be detrimental due to its pro-cancerous outcome. For example, while hyperactivation of stress responses like the HSR and UPR^ER^ can promote longevity in model organisms [1], but both HSF1 and XBP1 (regulator of UPR^ER^) have been linked to increased cancer pathology [8]. However, our study found that inhibiting BRD4 function destabilizes actin in senescent cells, resulting in loss of adherence and increased senescent cell death, which reveals a potential therapeutic avenue of targeting BRD4 as a senolytic. Radiation and chemotherapy are known to cause accumulation of senescent cells, including in non-cancerous tissues. The accumulation of senescent cells can promote tumor relapse and metastasis. Inhibition of BRD4 promotes both anti-cancer and senolytic outcomes, which provides the first potential drug target to tackle both problems simultaneously (Figure 1, right).

Perhaps the most critical future study for this potential avenue of therapeutic intervention is to better understand the impact of BRD4 inhibition on healthy, dividing cells. Alternatively, rather than targeting BRD4 directly, it would be of interest to identify specific transcriptional targets of BRD4, which could reveal additional and more directed therapeutic approaches. Overall, our study ascribes a novel function to BET-1/BRD4 in cytoskeletal maintenance, which opens up multiple exciting avenues of research investigating how BET-1 and BRD4 can impact stress resilience, aging, and age-related diseases.
